# Amlodipine and frusemide: pharmacological factors contributing to increased fall risk in concurrently treated patients – a retrospective cross-sectional study

**DOI:** 10.3389/fphar.2025.1598161

**Published:** 2025-07-28

**Authors:** Aymen Alqurain, Murtada Albaharnah, Samanah Al Zayer, Maha Alanzi, Razan Alblushi, Rawan Aleid, Rand Ashoor, Ali Albahrani, Mustafa Almahdi, Samaher Al-Shaibi, Luma Ameer, Sherihan Ghosn, Marwa Algoraini, Nawal Alsubaie, Afnan Alshnbari, Fadhel A. Alomar

**Affiliations:** ^1^Department of Clinical Practice, Faculty of Pharmacy, Northern Border University, Rafha, Saudi Arabia; ^2^Department of Pharmaceutical Care, King Fahad University Hospital, Imam Abdulrahman bin Faisal University, Al Khobar, Saudi Arabia; ^3^Department of Pharmaceutical Care, Mouwasat Hospital, Qatif, Saudi Arabia; ^4^Department of Pharmacy, Mohammed Al-Mana College for Medical Sciences, Dammam, Saudi Arabia; ^5^ Department of Pharmaceutical Care, RAM Clinics, Al Khobar, Saudi Arabia; ^6^ Department of Pharmaceutical Care, Mouwasat Hospital, Al Khobar, Saudi Arabia; ^7^School of Medicine, Dar Al Uloom University, Riyadh, Saudi Arabia; ^8^Department of Respiratory Therapy, Mohammed Al-Mana College for Medical Sciences, Dammam, Saudi Arabia; ^9^ Pharmacy Services Department, Johns Hopkins Aramco Healthcare (JHAH), Dhahran, Saudi Arabia; ^10^Foundation Year Department, Mohammed Al-Mana College for Medical Sciences, Dammam, Saudi Arabia; ^11^Department of Pharmacy Practice, College of Pharmacy, Princess Nourah bint Abdulrahman University, Riyadh, Saudi Arabia; ^12^ Department of Pharmacology, College of Pharmacy, Imam Abdulrahman Bin Faisal University, Dammam, Saudi Arabia

**Keywords:** amlodipine, frusemide, prescribing cascade, fall risk, orthostatic hypotension

## Abstract

**Background:**

Calcium channel blockers, such as amlodipine, are commonly prescribed for hypertension but can cause peripheral edema, often requiring adjunctive frusemide. Concerns exist regarding the potential increase in fall risk, particularly in older populations. However, few studies have assessed the prevalence of amlodipine and frusemide combination (AFC) prescriptions and their association with fall risk factors.

**Objectives:**

The aims of this study are to determine the prevalence of AFC prescriptions and evaluate their association with fall risk factors in an outpatient cardiology clinic population.

**Methods:**

This retrospective, cross-sectional study included patients aged ≥40 years from Al-Qatif Central Hospital’s outpatient cardiology clinic (January 2021 -December 2022) prescribed amlodipine. Fall risk factors were identified from literature. The Charlson Comorbidity Index (CCI) was used to estimate 1-year mortality risk. The number of prescribed orthostatic hypotension-inducing drugs (OHDs) and fall-risk increasing drugs (FRIDs) was recorded. Binary logistic regression was performed to determine the association between AFC prescriptions and fall risk factors, adjusting for significant covariates. Results are expressed as adjusted odds ratios (OR) with 95% confidence intervals (CI).

**Results:**

Of 3,681 patients, 18%. Were prescribed AFC. AFC patients were older (70 vs. 64 years, *P* < 0.001), had a higher prevalence of diabetes mellitus (64% vs. 44%, *P* < 0.001), anemia (55% vs. 32%, *P* < 0.001), and osteoporosis (51% vs. 28%, *P* < 0.001), and received more OHDs prescriptions (2.8 vs. 1.3, *P* < 0.001) compared to non-AFC patients. Higher CCI scores (OR = 1.51, 95% CI 1.41–1.62) and more OHDs prescriptions (OR = 2.5, 95% CI 2.3–2.7) were significantly associated with AFC prescriptions.

**Conclusion:**

AFC prescriptions are prevalent, and patients prescribed AFC have higher prevalence of fall risk factors. Comprehensive patients assessment is essential to minimize fall risk and related complications.

## 1 Introduction

The phenomenon of prescribing cascades, where additional medications are prescribed to manage adverse drug effects, is a growing concern, particularly in poly-medicated older population (Kalisch et al., 2011; Rochon and Gurwitz, 2017). One such cascade involves the concurrent prescription of amlodipine, a calcium channel blocker for hypertension and angina, and frusemide, a loop diuretic for fluid retention and edema (Savage et al., 2020; Woodford, 2020; Shahid et al., 2024). While this combination is common, concerns about its safety have been raised, particularly regarding increased fall risk due to its effects on blood pressure regulation and orthostatic hypotension (Savage et al., 2020; Ndai et al., 2024).

Amlodipine is linked to dose-dependent peripheral edema, a common adverse effect that often prompts the addition of frusemide (Shahid et al., 2024). However, studies suggest that diuretics may not effectively mitigate calcium channel blockers-induced edema, leading to unnecessary medication use and exposing patients to additional adverse drug reactions, such as dehydration, electrolyte imbalance, and hypotension (van Hamersvelt et al., 1996).

Therefore, amlodipine and frusemide combination (AFC) serves as a key example of a prescribing cascade that may inadvertently increase the risk of falls, particularly among older patients. Fall risk is multifactorial, influenced by factors such as advanced age, polypharmacy, chronic comorbidities, and medications that affect hemodynamic stability (Liu et al., 1995; Gale et al., 2016; Juraschek et al., 2017; Rashedi et al., 2019; Amano et al., 2024). Falls among older populations represent a major public health concern, as they are a leading cause of morbidity, disability, and mortality (Liu et al., 1995; Milos et al., 2014). Orthostatic hypotension-inducing drugs (OHDs) and fall-risk increasing drugs (FRIDs) are frequently implicated in fall-related hospitalizations (Milos et al., 2014; Al-Qurain et al., 2020). Given that both amlodipine and frusemide have hypotensive effects, their concurrent use requires careful assessment of fall risk, particularly in patients with additional predisposing factors.

Despite concerns regarding the safety of AFC prescriptions, limited research has explored the prevalence of this combination and its association with known fall risk factors. Understanding these associations is crucial for optimizing prescribing practices and minimizing unnecessary medication exposure, especially among poly-medicated older patients (Alqurain et al., 2024a). This study aims to determine the prevalence of AFC prescriptions in an outpatient cardiology clinic, identify the prevalence of key fall risk factors, including polypharmacy, comorbidities, and medication-related risks, among AFC users, and evaluate the association between AFC prescriptions and the presence of fall risk factors. By addressing these objectives, this study provides essential data that may inform safer prescribing practices and enhance fall prevention strategies for patients receiving AFC prescriptions.

## 2 Materials and methods

### 2.1 Study design, study population, and ethical considerations

This study is a secondary analysis of data collected retrospectively from patients medical records. The cross-sectional design was selected because both the exposure and the associated risk factors were assessed concurrently at the time of the patient’s initial recorded visit during the study period. No follow-up or longitudinal data were collected to assess outcomes over time. Data for this study were collected as per the approval from the Institutional Review Board (IRB) at Mohammed Al-Mana College for Medical Sciences (SR/RP/79, Approval Date 17 February 2022) and the IRB at the Qatif Central Hospital (QCH-SRECO 19-2022, Approval Date 8 June 2022). Data were collected between 1^st^ August 2022 and 20^th^ February 2023 from the hospital information technology department. All participants were deidentified, and a waiver of consent collection was approved by the IRB as data were collected retrospectively.

The study included patients aged 40 years or older who attended an outpatient cardiology clinic and were prescribed amlodipine between the period of January 2021 and December 2022. The age of 40 years was chosen as the inclusion criteria, as aging influences the epidemiology of multiple morbidities, often requiring the prescription of multiple drugs to manage these comorbidities, thereby increasing the risk of drug-drug interactions and adverse drug reactions (Divo et al., 2014). Patients younger than 40 years old, those attending other medical care or surgical care clinics, an emergency department, admitted to the hospital wards or intensive care units, and those not prescribed amlodipine or who visited the hospital outside the study period, were excluded. For patients with multiple visits during the study period, only data from their first reported visit were included.

### 2.2 Data collection, measures and definitions

Patients’ demographic data, comorbidities and recent laboratory findings were retrieved from the electronic medical record, while the prescribed medications were verified from the pharmacy electronic records. Comorbidities were identified and coded as per the International Classification of Diseases, 10th revision, 2016 (ICD-10) (World Health Organisation, 2016). Fall risk factors were identified based on literature and included selected comorbidities and medication profiles. Due to incomplete documentation, prior falls were not included in the analysis. Comorbidities associated with an increased risk of falls, such as anemia, diabetes mellitus, and osteoporosis were identified and documented based on established literature (Rashedi et al., 2019; Amano et al., 2024). The Charlson Comorbidities Index (CCI) was calculated to predict 1-year mortality risk, and the creatinine clearance (CrCl) was estimated using the Cockcroft-Gault Equation (Cockcroft and Gault, 1976; Charlson et al., 1987). Due to the inability to fully discriminate specific morbidities, osteoarthritis and rheumatoid arthritis were grouped under “arthritis-related diseases”, whereas different types of pain in the lower and upper back, muscles, bones, joints, ligaments, and tendons were grouped under “musculoskeletal pain” (Alqurain et al., 2024b).

The medications data, including long-term regular prescriptions, short-term, as-needed prescriptions, and supplements were collected and coded as per the Anatomical Therapeutic Chemical (ATC) classification system. The total number of prescribed medications (NPM), the total number of OHDs and FRIDs were counted and documented (Milos et al., 2014; Al-Qurain et al., 2020). Notably, for statistical analysis, “OHDs” was adjusted by excluding amlodipine and frusemide from the final count to avoid repeating variables in the binary regression.

Several situations were identified where the AFC prescription may not be considered a prescriber cascade, such as congestive heart failure, chronic kidney disease, or end-stage renal disease (Yancy et al., 2017). Frusemide is prescribed in these clinical situations to maintain euvolemia in patients at risk of hypervolemia (Vouri et al., 2018). Therefore, in the current study, AFC was defined as the initiation or continuation of frusemide in the absence of congestive heart failure, chronic kidney disease, or end-stage renal disease.

In the current study, two types of classification were used. In the first, patients were classified based on their age into two groups: middle-aged patients (<65 years) and older patients (≥65 years), to assess differences in prevalence pattern between these two cohorts. In the second, more detailed classification, patients were divided into six different groups (40–49, 50–59, 60–69, 70–79, 80–89, 90 years or older) to better examine trends in AFC prevalence across different age groups.

### 2.3 Statistical analysis

Demographic variables, comorbidities, and medication were summarized as follows: mean ± standard deviation (SD) for parametric continuous variables, median with interquartile range (IQR) for non-parametric continuous variables, and number with frequency (%) for binary variables. For comparisons of continuous variables, the Student’s *T*-test, and the Mann-Whitney *U* test were used for parametric and non-parametric data, respectively. The Chi-square test was used to compare the frequency of categorical variables between groups. Trends in AFC across different age groups were assessed using analysis of variance (ANOVA) test. Binary logistic regression was performed to compute unadjusted and adjusted odds ratios (OR) with 95% confidence interval (95% CI) to examine the association between the factors contributing to increased fall risk and AFC prescription within the cohort. The binary logistic regression was adjusted for covariates that either reached statistical significance at (*P* < 0.05) in univariate analysis or had clinically relevant to fall risk, as recommended by previous studies (Ranganathan et al., 2017). Multicollinearity was assessed using the variance inflation factor. Statistical analysis was performed using the SPSS statistical software package, version 23, and a *P* ≤ 0.05 was considered statistically significant.

## 3 Results

This study included 3,681 patients, with a prevalence of AFC prescription of 18%. Female patients were more frequent prescribed AFC compared to male patients (54% vs. 46%, *p* = 0.02) (Table 1). Patients prescribed AFC were older (70 vs. 64 years, *p* < 0.001), had a higher body weight (79 kg vs. 77 kg, *p* = 0.01), a lower average CrCl value (87 mL/min vs. 92 mL/min, *p* = 0.009) and a higher median CCI score (7 vs. 5, *p* < 0.001) compared to those not prescribed AFC. Interestingly, patients prescribed AFC were also more frequently prescribed OHDs and FRIDs compared to those not prescribed AFC (2.8 vs. 1.3, *p* < 0.001 and 0.7 vs. 0.5, *p* < 0.001 respectively) (Table 1). As shown in Table 1, patients prescribed AFC had a significantly higher prevalence of diabetes mellitus (65% vs. 44%, *p* < 0.001), anemia (55% vs. 32%, *p* < 0.001), musculoskeletal pain (65% vs. 57%, *p* = 0.001) and osteoporosis (51% vs. 38%, *p* < 0.001) compared to those not prescribed AFC. Table 1 shows that patients with AFC prescription had higher prevalence of hypertension (91% vs. 64%, *p* < 0.001), ischemic heart diseases (100% vs. 61%, *p* < 0.001) and arrhythmia (8% vs. 1%, *p* < 0.001) compared to those not prescribed AFC.

**TABLE 1:** Characteristics of the patients included in the study classified based on amlodipine and frusemide combination prescription.

Characteristics	Entire cohort	Prescribed amlodipine and frusemide	Prescribed amlodipine	*P*-value
	n = 3,681	n = 644	n = 3,037	
Age, mean (SD)	65 (14)	70 (13)	64 (14)	<0.001
Gender (female), n (%)	1842 (50)	350 (54)	1,492 (49)	0.02
(male), n (%)	1839 (50)	294 (46)	1,545 (51)	
Body weight (Kg), mean (SD)	78 (19)	79 (20)	77 (18.7)	0.01
CCI, median (IQR)	5 (4–7)	7 (6–9)	5 (4–7)	<0.001
NPM, mean (SD)	7 (6)	7 (6)	7 (6)	0.6
OHDs, mean (SD)	1.6 (1.5)	2.8 (1.9)	1.3 (1.3)	<0.001
FRIDs, mean (SD)	0.5 (1)	0.7 (1.1)	0.5 (0.9)	<0.001
CrCl (mL/min), Mean (SD)	91 (42)	87 (40.7)	92 (42)	0.009
Comorbidities				
Diabetes mellitus, n (%)	1745 (47)	421 (65)	1,324 (44)	<0.001
Anemia, n (%)	1,323 (36)	356 (55)	967 (32)	<0.001
Musculoskeletal pain, n (%)	2,160 (59)	416 (65)	1744 (57)	0.001
Arthritis related diseases, n (%)	2,477 (67)	435 (68)	2042 (67)	0.9
Osteoporosis, n (%)	1,482 (40)	331 (51)	1,151 (38)	<0.001
Hypertension, n (%)	2,519 (68)	585 (91)	1934 (64)	<0.001
Ischemic heart diseases, n (%)	2,500 (68)	644 (100)	1856 (61)	<0.001
Heart failure, n (%)	329 (9)	59 (9)	270 (9)	0.8
Arrhythmia, n (%)	83 (2)	48 (8)	35 (1)	<0.001

Abbreviations. CCI, Charlson comorbidity index; NPM, Number of prescribed medications; OHDs, Orthostatic hypotension inducing drugs; FRIDs, Falls risk increasing drugs, CrCl = Creatinine clearance.

**FIGURE 1:**
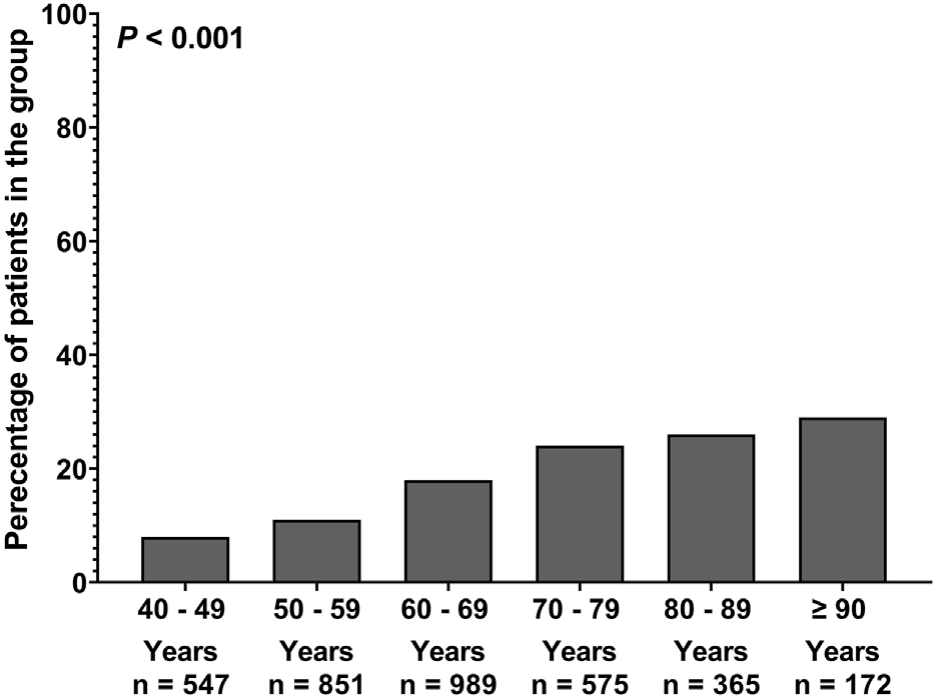
Prevalence of amlodipine and frusemide combination among different age groups.

Another analysis was conducted to determine the pattern of AFC occurrence across different age groups. Figure 1 illustrates a progressive increase in AFC occurrence with advancing age. Further analysis was conducted to assess the different characteristics between older vs. middle-aged patients. The analysis revealed that older patients were more likely to be female individuals (56% vs. 45%, *p* < 0.001), had a higher CCI score (7 vs. 4, *p* < 0.001) and had a lower CrCl value (73 vs. 108, *p* < 0.001) (Table 2). In addition, older patients presented with a higher prevalence of diabetes mellitus (52% vs. 43%, *p* < 0.001), anaemia (40% vs. 33%, *p* < 0.001), osteoporosis (44% vs. 37%, *p* < 0.001), hypertension (70% vs. 67%, *p* = 0.03), ischemic heart diseases (70% vs. 76%, *p* = 0.002), and arrhythmia (3% vs. 1%, *p* < 0.001) (Table 2). Interestingly, Table 2 shows that older patients were prescribed AFC more often compared to middle-aged patients (24% vs. 12%, *p* < 0.001).

**TABLE 2:** Characteristics of the patients included in the study classified based on patients aged.

Characteristics	Entire cohort	Older patients	Middle-aged patients	*P*-value
	n = 3,681	n = 1774	n = 1907	
Age, mean (SD)	65 (14)	76 (9)	54 (7)	<0.001
Gender (female), n (%)	1842 (50)	992 (56)	850 (45)	<0.001
(male), n (%)	1839 (50)	782 (44)	1,057 (55)	
Body weight (Kg), mean (SD)	78 (19)	74 (17)	81 (20)	0.3
CCI, median (IQR)	5 (4–7)	7 (6–8)	4 (3–5)	<0.001
NPM, mean (SD)	7 (6)	7 (6)	8 (6)	0.8
OHDs, mean (SD)	1.6 (1.5)	1.6 (1.6)	1.5 (1.5)	0.2
FRIDs, mean (SD)	0.5 (1)	0.5 (0.9)	0.5 (1)	0.9
CrCl (mL/min), Mean (SD)	91 (42)	73 (32)	108 (43)	<0.001
Amlodipine and frusemide combination, n (%)	644 (18)	419 (24)	225 (12)	<0.001
Comorbidities				
Diabetes mellitus, n (%)	1745 (47)	930 (52)	815 (43)	<0.001
Anemia, n (%)	1,323 (36)	700 (40)	623 (33)	<0.001
Musculoskeletal pain, n (%)	2,160 (59)	1,069 (60)	1,091 (57)	0.06
Arthritis related diseases, n (%)	2,477 (67)	1,196 (67)	1,281 (67)	0.9
Osteoporosis, n (%)	1,482 (40)	775 (44)	707 (37)	<0.001
Hypertension, n (%)	2,519 (68)	1,244 (70)	1,275 (67)	0.03
Ischemic heart diseases, n (%)	2,500 (68)	1,249 (70)	1,251 (66)	0.002
Heart failure, n (%)	329 (9)	143 (8)	186 (10)	0.07
Arrhythmia, n (%)	83 (2)	57 (3)	26 (1)	<0.001

Abbreviations. CCI, Charlson comorbidity index; NPM, Number of prescribed medications; OHDs, Orthostatic hypotension inducing drugs; FRIDs, Falls risk increasing drugs, CrCl = Creatinine clearance.

After adjusting for confounding covariates including age, body weight, sex, CCI, CrCl, OHDs and FRIDs, logistic regression analysis demonstrated that AFC prescription was positively associated with increasing body weight (OR = 1.02, 95% CI 1.01–1.024), higher CCI score (OR = 1.5, 95% CI 1.4–1.6), and greater OHDs prescription (OR = 2.5, 95% 2.3–2.7), while it was negatively associated with increasing FRIDs prescription (OR = 0.8, 95% CI 0.7–0.9) (Table 3).

**TABLE 3:** Association between patients’ characteristics and the occurrence of amlodipine and frusemide combination among the cohort.

	Odds ratio	95% confidence interval	*P*-value
Age	1	0.9–1.02	0.5
Body weight (kg)	1.02	1.01–1.024	<0.001
Gender (female)	1.2	0.9–1.5	0.2
Charlson comorbidity index	1.5	1.4–1.6	<0.001
Creatinine clearance (min/mL)	1	0.99–1.004	0.9
Orthostatic hypotension inducing drugs prescription	2.5	2.3–2.7	<0.001
Fall risk increasing drugs prescription	0.8	0.7–0.9	<0.001
Number of prescribed medications	0.99	0.98–1.01	0.5
Anemia	1.3	1.1–1.7	<0.001
Diabetes mellitus	0.87	0.7–1.1	0.2
Musculoskeletal pain	0.97	0.8–1.2	0.7
Arthritis related diseases	0.92	0.7–1.2	0.5

## 4 Discussion

This study highlights significant associations between the prescription of AFC and factors contributing to an increased risk of falls. The findings demonstrate that AFC prescriptions were prevalent among older adults and were strongly associated with higher CCI score, the use of OHDs prescriptions, and the presence of anaemia. Considering these associations, healthcare providers must carefully evaluate the appropriateness of AFC use in older patients, ensuring that the therapeutic benefits outweigh the potential risks while minimizing adverse drug reactions.

A key finding from this study is that 18% of the included cohort were prescribed AFC, a notably higher proportion compared to previously reported values. A study from the United States reported that 5%–12% of the cohort were exposed to and continued using AFC, whereas another study reported that about 3.5% of older patients with hypertension were prescribed AFC during their first year of therapy (Vouri et al., 2018; Ndai et al., 2024). The high prevalence reported in the current study highlights the importance of early detection of this combination to mitigate the risk of falls associated with orthostatic hypotension.

Another key finding is the significant association between increasing CCI scores and AFC occurrence. A higher CCI score reflects a greater burden of chronic illness, which inherently increases the risk of falls (Charlson et al., 1987; Milos et al., 2014; Rashedi et al., 2019). Additionally, due to the retrospective design of the current study, the CCI index was used as an alternative measure of frailty (Al-Qurain et al., 2021). Frail older patients exhibit a higher rate of falls, including recurrent falls compared to non-frail (Wong et al., 2015; Cheng and Chang, 2017). Recent studies have further demonstrated that frailty is associated with an increased risk of orthostatic hypotension and falls in older patients (Kocyigit et al., 2019; Shaw et al., 2019).

Another significant observation is the association between the prescription of OHDs and the occurrence of AFC. This finding is particularly relevant given that a recent study highlighted that OHDs prescriptions, including antihypertensives, increase the likelihood of polypharmacy by 20% among older patients (Alqurain et al., 2024a). Polypharmacy is a well-established risk factor for falls due to the increased potential for drug-drug interactions and adverse drug reactions (Alqurain et al., 2024a). Considering this association, routine medication reviews should prioritize minimizing unnecessary OHDs use, while maintaining optimal cardiovascular management (Scott et al., 2015; Zhou et al., 2017). Collaboration between pharmacists and healthcare providers is essential to evaluate whether alternative treatment strategies can be employed to reduce fall risk while achieving therapeutic goals.

Another important finding from the current study is the inverse association observed between FRIDs prescriptions and AFC use (OR = 0.8, 95% CI 0.7–0.9). This may reflect clinician-driven deprescribing behaviours, as prescribers intentionally avoid or reduce FRIDs use in patients already exposed to falls risk increasing drugs. This explanation is supported by previous study finding as prescribers often reduce or deprescribe medications potentiating risk of falls among vulnerable patients presented with polypharmacy and frailty (Reeve et al., 2014). Similarly, a clinical review reported that similar interventions are linked to reduced fall risk via medication review and deprescribing strategies (Van Poelgeest et al., 2021).

Furthermore, the study identified a high prevalence of diabetes mellitus, anaemia, and osteoporosis among AFC users, conditions that collectively increase the risk of falls. Diabetes mellitus contributes to fall risk through peripheral neuropathy and hypoglycaemia-related dizziness, while anaemia exacerbates fatigue and muscle weakness due to impaired oxygen delivery. (Stauder and Thein, 2014; Zhou et al., 2017; Rashedi et al., 2019; Amano et al., 2024). Osteoporosis further increases susceptibility to severe injuries following a fall, increasing morbidity and mortality risk (Avdic et al., 2004; Wang et al., 2021). The combination of AFC prescriptions and these comorbid conditions emphasizes the need for targeted fall prevention strategies, including regular screening for anaemia, careful monitoring of glucose levels, and interventions aimed at enhancing bone health in high-risk individuals.

Older patients are at an increased risk of lower extremity edema and prescribing amlodipine may further exacerbate this risk, potentially leading to the concurrent use of frusemide (Vouri et al., 2018; Ndai et al., 2024). Notably, this study identified a higher prevalence of AFC prescription among older patients. Advanced age is also associated with the presence of multiple morbidities, which often necessitate the use of multiple drugs, leading to polypharmacy (Al-Qurain et al., 2020). Therefore, healthcare providers should consider conducting comprehensive medication reviews to assess the risk of adverse drug reactions associated with AFC prescription among older patients, ensuring the effective management of hypertension while minimizing adverse drug reactions and drug-drug interactions (Al-Qurain et al., 2021).

Another finding from the current study is that age, gender, and body weight were significantly higher in the AFC group. This finding aligns with previously published studies where these characteristics were linked to increased risk of falls and falls related injuries among older population (Wei and Hester, 2014; Waters et al., 2019; Minhee et al., 2023). However, the regression model did not identify significant association between these characteristics and AFC prescribing. Instead, these variables were included in the multivariable regression model to control for their confounding effect. The associations observed between AFC prescribing and high CCI scores and OHD use suggest an independent link, supporting the need for caution in AFC prescribing irrespective of baseline demographic differences.

In the current study, patients with CHF, CKD, and ESRD were excluded from the definition of AFC to reduce misclassification because the retrospective design precludes confirmation of prescribing intent. Therefore, we cannot definitively confirm whether frusemide was added to manage amlodipine-induced oedema. To address this, a sensitivity analysis excluding patients with liver cirrhosis, a potential alternative indication for diuretics, was conducted and the results were consistent with the early findings. However, prospective studies are needed to accurately establish causality and sequence of prescribing decisions.

This study has several notable strengths. The current study is the first of its kind to report on the prevalence of AFC prescriptions and evaluate factors contributing to increased fall risk among patients attending a cardiology clinic in the Eastern Region of Saudi Arabia. Specifically, amlodipine and frusemide are common medications prescribed for older populations and their use can be defined as prescriber cascade in some cases which is an important medication related problem especially in the geriatric medicine. Additionally, the study benefits from a large sample size, providing robust statistical power to detect associations between AFC prescription and the identified risk factors. The comprehensive analysis, incorporating binary linear regression, allows for a more thorough assessment of the factors contributing to increased fall risk associated with AFC prescription. Furthermore, the study explores differences in AFC prescription patterns between middle-aged and older patients, which could help develop strategies for detecting the phenomenon and mitigating fall risk in different patient groups.

However, several limitations should be acknowledged. As retrospective cross-sectional study, it was difficult to accurately identify the phenomenon of prescribing cascade owing to the inability to determine the exact initiation date of the prescribed medications. To address this, the study investigated the concurrent prescription of amlodipine and frusemide as an indicator for the occurrence of this phenomenon (Vouri et al., 2018; Savage et al., 2020). Another limitation is the lack of information regarding the duration of AFC prescription. Due to the retrospective design and the nature of electronic health records used in this study, we were unable to determine whether the prescriptions were chronic or short-term. As such, the AFC exposure was assessed only as a binary variable, and the impact of duration on fall risk could not be evaluated. In addition, the cross-sectional design limited the ability to assess the appropriateness of medications and to interpret adverse drug reactions more broadly. Another limitation is the absence of body mass index data due to incomplete height documentation in the medical records, which precluded the assessment of obesity status. While body weight was included in the analysis, it may not fully capture obesity-related risk. Frailty was not directly measured in this study due to the retrospective nature of data collection. While CCI was used as a proxy indicator of frailty-related burden (Charlson et al., 1987), we recognize that it is primarily a mortality risk tool and not a validated frailty assessment instrument, but was used to estimate frailty status based on the patients’ current disease states. Future prospective studies should incorporate structured frailty tools for more accurate risk stratification. The Lack of follow-up data further limited the understanding of longer-term treatment outcomes, including efficacy, adverse drug reactions, and readmission rates. Lastly, due to its cross-sectional in nature, this study did not allow for an examination of the trajectory of AFC prescription over time or their associations with changes in hypertension and fall incidence trajectories.

Based on our results, interventions should be implemented to increase healthcare providers’ awareness of the potential fall risk associated with AFC prescriptions. Such interventions could include educational programs, clinical guidelines, or decision-support tools to equip healthcare professionals with the necessary knowledge to identify and prevent unnecessary prescribing cascades in these patients.

The phenomenon of prescribing cascade has gained attention within geriatric medicine as a significant contributor to the global challenge of polypharmacy (Brath et al., 2018). Therefore, early detection and assessment of AFC prescription are a crucial component of comprehensive care for these patients (Scott et al., 2015). It is recommended that patients at risk of falls associated with AFC prescription be closely monitored to ensure appropriate management of blood pressure and prevention of adverse drug reactions.

## 5 Conclusion

AFC prescriptions remain an ongoing concern, with 18% of the cohort being prescribed this combination. Older age, diabetes mellitus and anemia were more common among patients prescribed AFC. AFC prescriptions were associated with OHDs prescriptions and increasing CCI score. This study highlights the importance of comprehensive patient assessment to minimize fall risk and related complications among patients prescribed AFC.

## Data Availability

The raw data supporting the conclusions of this article will be made available by the authors, without undue reservation.
