# Supplementary material for: Amlodipine and frusemide: pharmacological factors contributing to increased fall risk in concurrently treated patients – a retrospective cross-sectional study
Source: Front Pharmacol. 2025 Jul 28;16:1598161. doi: 10.3389/fphar.2025.1598161 (PMC12336181; doi:10.3389/fphar.2025.1598161)
Supplement: Supplementary file 1 [file Supplementaryfile1.docx]

**Supplementary Materials**

Table S1: Fall risk-increasing drugs and drugs that may cause or worsen orthostatic blood pressure according to the list from the Swedish National Board of Health and Welfare

| ATC code | Drugs/group of drugs |
| --- | --- |
| Increase the fall risk (FRIDs) |  |
| NO2A | Opioids |
| N05A (NO5AN excluded) | Antipsychotics (lithium excluded) |
| N05B | Anxiolytics |
| N05C | Hypnotics and sedatives |
| N06A | Antidepressants |
| May cause or worsen Orthostatism (OHDs) |  |
| C01D | Vasodilators used in cardiac diseases |
| C02 | Antihypertensives |
| C03 | Diuretics |
| C07 | Beta blocking agents |
| C08 | Calcium channel blockers |
| C09 | Renin-angiotensin system inhibitors |
| G04CA | Alpha-adrenoreceptor antagonists |
| N04B | Dopaminergic agents |
| N05A (NO5AN excluded) | Antipsychotics (lithium excluded) |
| N06A | Antidepressants |

ATC: Anatomical Therapeutic Chemical classification system.

**Table S2** Prevalence of specific FRIDs and OHDs prescribing among the cohort classified based on AFC prescription.

| Characteristics | Entire cohort | Prescribed amlodipine and frusemide | Prescribed amlodipine | *P*-value |
| --- | --- | --- | --- | --- |
|  | *n = 3681* | *n = 644* | *n = 3037* |  |
| FRID |  |  |  |  |
| Opioids, n (%) | 609 (17) | 110 (17) | 499 (16) | 0.7 |
| Antipsychotics, n (%) | 70 (2) | 11 (2) | 59 (2) | 0.7 |
| Anxiolytics, n (%) | 137 (4) | 20 (3) | 117 (4) | 0.4 |
| Antidepressants, n (%) | 171 (5) | 40 (6) | 131 (4) | 0.04 |
| OHDs |  |  |  |  |
| ACEI/ARBs, n (%) | 353 (10) | 52 (8) | 301 (10) | 0.2 |
| CCBs, n (%) | 319 (9) | 644 (100) | 3037 (100) | Na |
| Diuretics (excluding frusemide), n (%) | 212 (6) | 34 (5) | 178 (6) | 0.6 |
| Anti-hypertensives, n (%) | 41 (1) | 8 (1) | 33 (1) | 0.7 |
| Beta blockers, n (%) | 324 (9) | 59 (9) | 265 (9) | 0.7 |
| Nitrates, n (%) | 30 (1) | 4 (0.6) | 26 (1) | 0.6 |
| Antidepressants, n (%) | 171 (5) | 40 (6) | 131 (4) | 0.04 |
| Antipsychotics, n (%) | 70 (2) | 11 (2) | 59 (2) | 0.7 |

**Table S3** Characteristics of the patients who prescribed amlodipine and frusemide combination classified based on their gender.

| Characteristics | Prescribed amlodipine and frusemide | Female patients | Male patients | *P*-value |
| --- | --- | --- | --- | --- |
|  | *n = 644* | *n = 350* | *n = 294* |  |
| Age, mean (SD) | 70 (13) | 72 (13) | 67 (13) | < 0.001 |
| Body weight (Kg), mean (SD) | 79 (20) | 77 (19) | 82 (21) | 0.006 |
| CCI, median (IQR) | 7 (6 – 9) | 8 ( | 7 (6 – 8) | < 0.001 |
| NPM, mean (SD) | 7 (6) | 7 (6) | 7 (6) | 0.6 |
| OHDs, mean (SD) | 2.8 (1.9) | 2.8 (1.9) | 2.9 (1.8) | 0.7 |
| FRIDs, mean (SD) | 0.7 (1.1) | 0.8 (1.2) | 0.6 (1) | 0.01 |
| CrCl (mL/min), Mean (SD) | 87 (40.7) | 75 (33) | 101 (45) | < 0.001 |
| Comorbidities | | | | |
| Diabetes mellitus, n (%) | 421 (65) | 231 (66) | 190 (65) | 0.7 |
| Anemia, n (%) | 356 (55) | 207 (59) | 149 (51) | 0.03 |
| Musculoskeletal pain, n (%) | 416 (65) | 247 (71) | 169 (58) | 0.001 |
| Arthritis related diseases, n (%) | 435 (68) | 239 (68) | 196 (67) | 0.7 |
| Osteoporosis, n (%) | 331 (51) | 205 (59) | 126 (43) | < 0.001 |
| Hypertension, n (%) | 585 (91) | 320 (91) | 265 (90) | 0.6 |
| Ischemic heart diseases, n (%) | 644 (100) | 350 (100) | 294 (100) | Na |
| Heart failure, n (%) | 59 (9) | 30 (9) | 29 (10) | 0.6 |
| Arrhythmia, n (%) | 48 (8) | 29 (8) | 19 (7) | 0.4 |

**Abbreviations.** *CCI = Charlson comorbidity index, NPM = Number of prescribed medications, OHDs = Orthostatic hypotension inducing drugs, FRIDs = Falls risk increasing drugs, CrCl = Creatinine clearance*.

**Table S4** Characteristics of the patients who prescribed amlodipine and frusemide combination classified based on their age.

| Characteristics | Prescribed amlodipine and frusemide | Older patient | Middle-aged patient | *P*-value |
| --- | --- | --- | --- | --- |
|  | *n = 644* | *n = 419* | *n = 225* |  |
| Age, mean (SD) | 70 (13) | 77 (9) | 56 (7) | < 0.001 |
| Gender (female), n (%) | 350 (54) | 249 (59) | 101 (45) | < 0.001 |
| (male), n (%) | 294 (46) | 170 (41) | 124 (55) |  |
| Body weight (Kg), mean (SD) | 79 (20) | 77 (20) | 83 (21) | 0.002 |
| CCI, median (IQR) | 7 (6 – 9) | 8 (7 – 9) | 6 (5 – 8) | < 0.001 |
| NPM, mean (SD) | 7 (6) | 7 (6) | 6 (6) | 0.2 |
| OHDs, mean (SD) | 2.8 (1.9) | 2.7 (1.9) | 3.1 (1.9) | 0.001 |
| FRIDs, mean (SD) | 0.7 (1.1) | 0.7 (1.1) | 0.7 (1.2) | 0.2 |
| CrCl (mL/min), Mean (SD) | 87 (40.7) | 75 (34) | 109 (43) | < 0.001 |
| Comorbidities | | | | |
| Diabetes mellitus, n (%) | 421 (65) | 278 (66) | 143 (64) | 0.5 |
| Anemia, n (%) | 356 (55) | 224 (54) | 132 (59) | 0.2 |
| Musculoskeletal pain, n (%) | 416 (65) | 272 (65) | 144 (64) | 0.8 |
| Arthritis related diseases, n (%) | 435 (68) | 285 (68) | 150 (67) | 0.7 |
| Osteoporosis, n (%) | 331 (51) | 210 (50) | 121 (54) | 0.4 |
| Hypertension, n (%) | 585 (91) | 379 (91) | 206 (92) | 0.6 |
| Ischemic heart diseases, n (%) | 644 (100) | 419 (100) | 225 (100) | Na |
| Heart failure, n (%) | 59 (9) | 40 (10) | 19 (8) | 0.6 |
| Arrhythmia, n (%) | 48 (8) | 33 (8) | 15 (7) | 0.6 |

**Abbreviations.** *CCI = Charlson comorbidity index, NPM = Number of prescribed medications, OHDs = Orthostatic hypotension inducing drugs, FRIDs = Falls risk increasing drugs, CrCl = Creatinine clearance*.
